# Improving machine learning models through explainable AI for predicting the level of dietary diversity among Ethiopian preschool children

**DOI:** 10.1186/s13052-025-01892-1

**Published:** 2025-03-24

**Authors:** Gizachew Mulu Setegn, Belayneh Endalamaw Dejene

**Affiliations:** 1 Department of Computer Science, Debark University, Debark, 90 Ethiopia; 2 University of Gondar, Gondar, 196 Ethiopia

**Keywords:** Dietary diversity, Ethiopia, Explanatory AI, Food group Predicting level, Preschool children, Machine learning algorithm

## Abstract

**Background:**

Child nutrition in Ethiopia is a significant concern, particularly for preschool-aged children. Children must have a varied diet to ensure they receive all the essential nutrients for good health. Unfortunately, many children in Ethiopia lack access to a range of foods, which can lead to malnutrition and other health issues. While machine learning (ML) has the potential to analyse extensive datasets, the lack of transparency in these models can impede their effectiveness in real-world applications, especially in public health. This research aims to enhance machine learning models by integrating Explainable AI (XAI) methods to more accurately predict the level of dietary diversity in Ethiopian preschool children.

**Methods:**

To Improve the ML Model for Predicting the Level of Dietary Diversity among Ethiopian Preschool Children. We employed an ensemble ML approach with XAI. The Ethiopian demographic health survey collected a dataset consisting of dietary information and relevant socioeconomic variables. The data were preprocessed to obtain quality data that are suitable for the ensemble ML algorithms to develop a model. We applied filter (chi-square and mutual information) and wrapper (sequential backwards) feature selection methods to identify the most influential factors for dietary diversity (DD). Ethiopia demographic health survey (from 2011 to 2019). Datasets were used. We developed a predictive model using a decision tree, random forest, gradient boosting, light gradient boosting, CatBoost, and XGBClassifier. We evaluated it using accuracy, precision, recall, F1_score, and receiver operating characteristic (ROC)-based evaluation techniques.

**Results:**

The ensemble ML models exhibited robust predictive performance, and light gradient boosting outperformed the other ensemble ML algorithms by 95.3%. The explainability of the Light Gradient Boosting Ensemble Model was determined using Eli5 and LIME. The child’s age, household wealth index, household region, source of drinking water, frequency of listening to the radio, and mother’s education level were the most crucial variables for the prediction of Minimum Dietary Diversity (MDD) in Ethiopia.

**Conclusions:**

The research effectively demonstrated that integrating Explainable AI with machine learning can accurately predict dietary diversity in preschoolers in Ethiopia. The results of this study have significant implications for stakeholders in child development and nutrition, as well as for policymakers and medical experts. Targeted interventions and policies to enhance the nutritional health of Ethiopian preschool children are made possible by the explainable AI model that has been constructed.

**Trial registration:**

Retrospectively registered.

## Introduction

“Dietary diversity” refers to consuming various food types at various times, including cereals, fruits, vegetables, meat, dairy products, and tubers. Ensuring suitable dietary intake can be achieved by including a wider range of foods and food groups in one’s diet [1]. Malnutrition continues to be an important issue for global public health [2, 3]. Globally, 10.9 million deaths (60% of all deaths) occurred in under 5 years. Inadequate feeding practices result in the deaths of approximately 3.4 million children under the age of five every year. Inadequate feeding practices during the first two years of life were the cause of 66% of these deaths [4]. Childhood malnutrition, which is closely linked with mortality and morbidity, is mostly caused by inadequate dietary diversity in developing countries [5, 6]. More chronically malnourished children worldwide reside in South Asia and sub-Saharan Africa than in any other part of the world [2]. The overall minimal dietary diversity (MDD) prevalence across East Africa was 10.4%, with Ethiopia (6.81%) and Rwanda (16.22%) having the lowest and highest rates, respectively, among preschool children [4]. By 2030, the Sustainable Development Goals (SDGs) of the United Nations aim to enhance the health and well-being of every child by eliminating preventable deaths among infants and children under the age of five. Nutritional objectives must be given top priority in the SDGs to accomplish this goal [7]. A summary of a household’s socioeconomic position and food access based on the last 24 h is given by the household dietary diversity score (HDDS) indicator. When a home consumes less than or equal to three food groups in the 24 h before the survey, it is said to have low household dietary diversity. The term “medium household dietary diversity” describes a household’s consumption of four to six food groups in the 24 h before the survey. Households with seven or more food groups consumed in the 24 h before the survey are considered to have a high level of dietary diversity [8, 9]. Increased nutrient intake and a lower risk of malnutrition are linked to a variety of food categories consumed. To achieve adequate nutrition and health results, households require an adequate variety of diets [10]. Several studies have been conducted to comprehend the determinants of dirty diversity in preschool children in Ethiopia. Previous studies on the determinants of inadequate dietary diversity have been conducted. In Ethiopia [2], only 8.5% of children achieved the recommended minimal level of dietary diversity. There was a significant association between MDD and maternal education, maternal income index, age of the child, and number of children under five years of age. In Addis Ababa, Ethiopia, cross-sectional data were used for the analysis, and 59.8% of the children had MDD (< 4 food groups). Children’s dietary diversity is positively associated with maternal education and household wealth status [11]. Other studies in Dire Dawa City, Eastern Ethiopia, were community-based cross-sectional studies, and the overall prevalence of MDD was 24.4%. Maternal education decision-making, antenatal care, postnatal care, and facility delivery were maternal factors. Moreover, the child’s age and sex are infant factors [6]. Another study in Ethiopia was a community-based cross-sectional study with a binary logistic regression at the kebele level in Chelia, Ethiopia, to identify significant factors from April 12 to April 30, 2020. Less than one-quarter (17.32%) of infants and young children aged 6 to 23 months had MDD, and having children aged 18–23 months, mothers aged 35–44 years, housewives as household heads, children of smaller family sizes, and caregivers who studied grades 9–12, who received information about food diversity during antenatal care and postnatal care visits, who travelled less than one hour to reach the market, and who had a high family income were significantly associated with having MDD [12]. Previous research, including [2, 6, 11–15], was conducted using cross-sectional methods that showed that the consumption of food, nutritional knowledge, attitudes, and sociodemographic variables are important factors that impact the achievement of dietary diversity, and only traditional descriptive statistical techniques have been applied to understand the factors influencing the diversity of diets. There is often a need for help in capturing the complex relationships and interactions among various factors, the absence of a nationwide study to obtain an in-depth understanding of the DD to develop evidence-based decisions or policies in Ethiopia, and a lack of studies that explore the effectiveness of ensemble ML algorithms and XAI tools, specifically for predicting dietary diversity among preschool children in Ethiopia. Machine learning approaches allow the development of an accurate model that can be applied to activities such as estimation, prediction, classification, or any other similar task [16]. Explanatory AI has shown potential for accurately predicting and proving more transparent and understandable for users by providing explanations for their decisions in addition to accurate predictions [17, 18]. This study aims to integrate ensemble machine learning and explainable AI techniques to improve the interpretability and accuracy of preschool children’s dietary diversity predictions to make understanding and interpretability easier for people. Therefore, this nationwide study was conducted:

- To evaluate the underlying structure and evolution of dietary diversity among preschool children in Ethiopia over time.
- To assess various machine learning algorithms using model evaluation metrics.
- This distinctive study employed XAI techniques, such as LIME and ELI5, to elucidate the overall behaviour of the model, identify significant features contributing to dietary diversity prediction models, and interpret the predictions generated by the model.

The results of this study will be useful for national-level policymaking, nutritionists, and organizations working toward improving nutrition interventions. Explainable AI techniques improve transparency in machine learning models, enabling stakeholders to comprehend predictions and make informed decisions. Addressing dietary diversity among preschool children in Ethiopia tackles a critical public health issue, vital for cognitive and physical development. Adapting the model to the Ethiopian context takes local factors into account, enhancing its applicability and relevance. This study advances the field of Explainable AI by applying it to a real-world public health challenge, potentially inspiring further research in other areas.

## Materials and methods

**Fig. 1:**
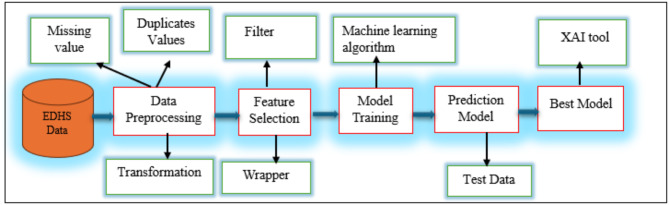
Pictorial representation of the research workflow

.In this section, we outline the overall workflow and methodology of the research, detailing the steps involved in this study. Figure 1 presents a visual representation of the experimental setup for predicting dietary diversity among preschool children in Ethiopia.

### Data description

In this study, we obtained data from the Ethiopia Demographic Health Survey (EDHS), which includes demographic information, socioeconomic factors, and food consumption patterns as illustrated in Table 1. The DHS serves as an open platform for sharing data across crises and organizations, facilitating the easy discovery and utilization of sociodemographic data for analysis [19]. The degree of DD was calculated based on the quantity of food consumed in 24 h. According to WHO guidelines, the level of DD was calculated using eight food groups, which included cereal, roots, and tubers; legumes and nuts; dairy products (cheese, yoghurt, other milk products); flesh foods (meat, fish, poultry, and organ meats); eggs; vitamin-A-rich fruits (mango, papaya, orange, avocado, banana, pineapple); any other fruits; and vitamin-A-rich vegetables (leaf, leaf of pumpkin, cabbage, lettuce) [1]. The DD level was calculated based on the preceding 24 h. The raw data includes a total of 28 attributes (with the DD level as the target class) and 28,047 instances before applying the synthetic minority oversampling technique (SMOTE). To classify preschool children’s DD, the final target variable was assigned “1” for high DD (> 4 food groups) and “0” for minimal DD (< 4 food groups) for the ensemble machine learning algorithm and XAI tools.

**Table 1:** Sociodemographic characteristics of preschool children (6–59 months) in Ethiopia

Variable	Category
Sex of child	Male
	Male
Mother’s age	15–19 years
	20–24 years
	25–29 years
	30–34 years
	35–39 years
	≥ 40 years
Child’s age	6–12 months
	13–24 months
	25–36 months
	37–48 months
	49–59 months
Birth order	First
	Second
	Third and above
Mother’s marital status	Married
	Widowed
	Single
	Divorced
Mother’s religion	Orthodox
	Catholic
	Protestant
	Muslim
	Others and Traditional
Mother’s education	No formal education
	Primary education
	Secondary education
	College and above
Mother’s occupation	Unemployed
	Employed
Wealth Index	Poorest
	Poor
	Middle
	Rich
	Richest
Place of residence	Rural
	Urban
Body mass index	Underweight
	Normal
	Overweight
	Obesity
Husband education level	No formal education
	Primary education
	Secondary education
	College and above
Type of cooking	Modern
	Traditional
Child is Twin	Yes
	No
Smoking cigarettes	Yes
	No
Contraception method	Traditional
	Modern
Sex of house Household Head	Male
	Female
Ever took an alcoholic drink?	No
	Yes
frequency of listening to Radio	Not at all
	Sometimes
	Almost Every day
The frequency of listening to Television	Not at all
	Sometimes
	Almost Every day
Frequency of Reading Newspaper	Not at all
	Sometimes
	Almost Every day
Region	Tigray, Afar, Amhara, Oromia, Somali, Benishangul-Gumuz, Southern Nations, Nationalities and Peoples Region (SNNPR), Gambella and Harari, Addis Ababa, and Dire Dawa
Source of drinking water	Unimproved
	Improved
Currently Breastfeeding	Yes
	No
*Health insurance*	Yes
	No
*Antenatal visits*	No antenatal visits or one-time
	Two time
	Three and above
*Presence of diarrhea*	Yes
	No

### Data preprocessing

To ensure data quality and consistency, the data were preprocessed to address cleaning and missing value imputation methods (modes for categorical data and means for continuous data) **(**Table 2**).** Table 2 illustrate the dataset used in this study with missing value. The validity of the dataset is frequently compromised by the presence of duplicate rows, which can lead to inaccurate analysis. In this paper, duplicate entries in the dataset were removed, and data transformation along with class imbalance adjustments were conducted. Some features had numerous distinct values and required transformation for mining purposes; for instance, features with multiple categorical values, such as the source of drinking water, body mass index, and wealth index, were converted into discrete values using binning discretization techniques.

**Table 2:** Missing value distribution

Missing Attribute	Number of Missing Values	Percentage (%)
legumes and nuts	16,405	58.5%
Antenatal visits	10,685	38.1%
Health insurance	10,654	38%
Source of drinking water	10,646	38%
Region	2606	9.3%
Body mass index	651	2.3%
Type of cooking	460	1.6%
Mother’s occupation	288	1%
Mother’s religion	248	0.9%
Husband education level	217	0.8%

The dataset is balanced to prevent bias toward any one class, which makes it easier to train the model. There are two types of approaches for data balancing: undersampling and oversampling. Undersampling can reduce model runtime and is easy to implement, but it has several disadvantages. Removing data from the original dataset may cause significant data loss. Overfitting is another possibility when there is not enough data. As a result, this study recommended oversampling. We utilized the SMOTE to balance the training dataset data. SMOTE has been shown to increase the accuracy of classification for resampling imbalanced datasets [20, 21].

### Feature selection and data splitting

The most important variables influencing the dietary diversity of households were determined through the use of feature selection approaches. By using these techniques, we could narrow down the variables that have the greatest impact on the results and concentrate our predictive model on the most important features. There are three main approaches for selecting features: the embedded, filter, and wrapper methods [22]. Filter Methods (Mutual Information, Chi-square Test) and Wrapper Methods (Step Backward Feature Selection) were applied to reduce features that were not necessary [27] as illustrated in Table 3. After feature selection, the data are split into training and testing sets of 80% and 20%, respectively.

**Table 3:** Important features based on feature selection methods

Methods Used	Features	Accuracy
Mutual Information	‘Age of child’, ‘Birth order’, ‘Mother education Level’, ‘Mother occupation’, ‘Wealth Index’, ‘Place of Residence’, ‘Body mass index’, ‘Type of Cooking ‘, ‘Type of Cooking’, ‘Contraception Method’, ‘husband Education Level’,' Frequency of listening Radio ‘,' Frequency of listening Television’, ‘Region ‘, ‘Frequency of Reading Newspaper’,' ‘currently breastfeeding’.	97.8**%**
Chi-square Test	‘Age of child’, ‘Birth order’, ‘Mothers Education Level’, ‘Mothers Occupation’, ‘Wealth Index’, ‘Place of Residence’, ‘Body mass index’, ‘Type of Cooking ‘, ‘Contraception Method’, ‘husband Education Level’,' Frequency of listening Radio ‘,' Frequency of listening Television ‘, ‘Frequency of Reading Newspaper’, Region, ‘’currently breastfeeding’	98.8**%**
Step Backward Feature Selection	Sex of child’, ‘age in 5-year group’, ‘Age of child’, ‘Religion’, ‘Mother education Level’, ‘Mother occupation’, ‘Wealth Index’, ‘body mass index’, ‘Contraception Method’. ‘Husband Education Level’.' ‘Frequency of listening to Radio’, ‘Region ‘, ‘currently Breastfeeding’, ‘Source of Drinking Water’, ‘Presence of diarrhea’	99.3**%**

**Fig. 2:**
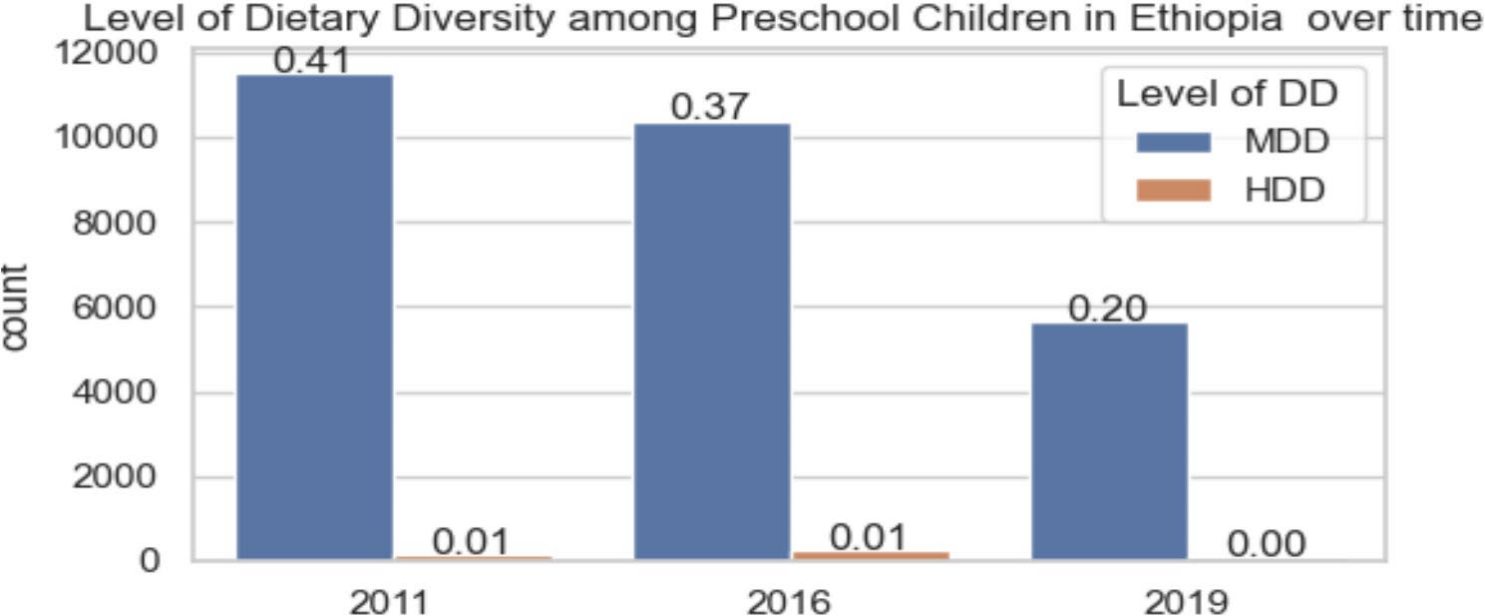
The level of dietary diversity among preschool children over time in Ethiopia

The incidence of MDD among preschool children in Ethiopia has decreased over time. This decline is attributed to mothers living far from health centers having greater odds of providing their children with a diversified diet, limited awareness of proper nutrition techniques, or inadequate access to nutritious foods. Family literacy impacts the dietary intake of preschool children, and a significant number of families in the area may contribute to MDD. However, due to the COVID-19 pandemic, the amount of data collected in 2019 was half that of previous years, as illustrated in Fig. 2.

## Result

The overall prediction performances of the DD models for preschool children are illustrated in Table 4. The model is trained after the data are ready. This involves incorporating the training data into the model and allowing it to develop a prediction model using the data provided [23]. In this study, we used six ML algorithms (Decision Tree, Random Forest, Gradient Boosting, Light Gradient Boosting, CatBoost, and XGBClassifier) to predict the DD of preschool children using features resulting from step-backward feature selection methods. Each model is trained and evaluated using training and testing sets. Each model’s performance was assessed using appropriate evaluation metrics [28], such as accuracy, precision, and recall; F1 scores; and ROC curves. In addition to determining how well the suggested approach works, this study also provides insight into how successfully ensemble machine learning algorithms and XAI tools can predict the DD of preschool children.

**Table 4:** The prediction performance of different machine learning models for the level of DD in preschool children using EDHS data

Model	Accuracy	Recall	Precision	F1-Score	ROC
Decision Tree	83.9%	83.9%	84.3%	84.0%	92.0%
Random Forest	82.2%	82.2%	82.7%	80.3%	91.0%
Gradient Boosting	92.6%	91.6%	91.7%	91.6%	98.0%
Light Gradient Boosting	95.3%	95.3%	95.5%	95.3%	99.0%
CatBoost	92.8%	91.7%	92.1%	91.9%	98.0%
XGBClassifier	83.8%	83.8%	84.3%	84.0%	92.0%

In conclusion, light gradient boosting stands out as the most effective homogeneous ensemble machine learning algorithm for predicting preschool DD levels based on EDHS data, achieving an accuracy of 95.3%. However, it remains unclear which sociodemographic features such as the child’s age, birth order, marital status, maternal education, region, frequency of newspaper reading, and breastfeeding significantly contributed to the overall performance of the ML algorithms in predicting preschool children’s DD. Consequently, the Light Gradient Boosting algorithm has been chosen for further exploration using the XAI tool.

### Explanations of ML models

Explainable artificial intelligence models can offer insights into how various factors and levels of dietary diversity in households are interconnected. These models may reveal significant features that clarify why the degree of dietary diversity varies among households. This XAI tool empowers policymakers and researchers to comprehend the underlying causes of dietary patterns. We employed XAI tools like Eli5 and LIME to ascertain how a model predicts outcomes and to elucidate how attributes influence those predictions. As shown in Table 5, the Eli5 model is utilized to extract the key features. Local contributions toward predicting the level of preschool DD are made using the RF model. The significance is indicated in the contribution column of the table. The test instance was fed into the trained ML model, which classified the instance as class 0 or class 1. According to the LightGBM model using Eli5, the wealth index, region, child’s age, maternal age, child’s sex, and maternal occupation are the most significant contributing features for DD. The contributions of these six features are 0.1517, 0.1575, 0.1020, 0.1136, 0.0251, and 0.0286, respectively. The Eli5 methodology for CatBoost was not applied because CatBoost estimators do not support the Eli5 XAI tool.

**Table 5:** Explanation of the local contribution of sociodemographic features through the Eli5 model in predicting the level of preschool DD in a single test instance using the LightGBM model. The presence of a question mark in a table generated by LIME may signify uncertainty or a need for further clarification regarding the results or interpretations provided

y = 0 (probability 0.995, score -5.310) top features		
Contribution?	**Feature**	**Value**
+ 1.613	Wealth Index	0.1517
+ 0.986	Region	0.1575
+ 0.565	Age of child	0.1020
+ 0.338	Age in 5-year group	0.1136
+ 0.141	Sex of child	0.0251
+ 0.099	Mother Occupation	0.0286
+ 0.090	Frequency of listening to Radio	0.0536
+ 0.073	Contraception Methods	0.0296
+ 0.061	Husband Education Level	0.0695
+ 0.019	Mother Education Level	0.0607
+ 0.007	Breastfeeding	0.0345
-0.046	Body Mass Index	0.0586
-0.118	Mother Religion	0.0643
-0.179	Source of Drinking Water	0.0333

### Model explanation using LIME with LightGBM model

**Fig. 3:**
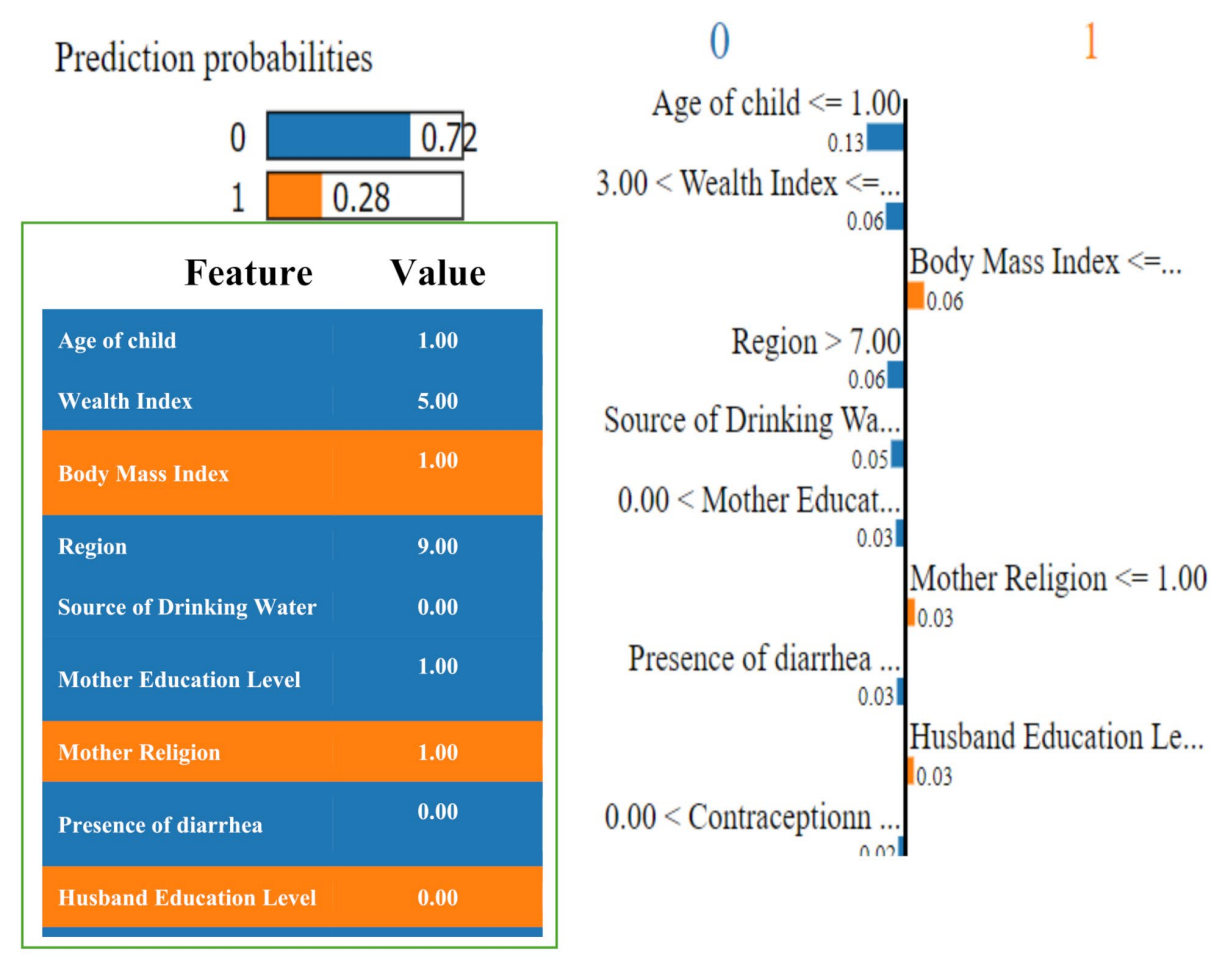
Illustrates the local contribution of sociodemographic features using the LIME model in classifying a single test instance (predicted class = MDD (0)) using the LightGBM model. The pink-marked cells represent the features that contributed most to classifying preschool children to HDD (1)

**Fig. 4:**
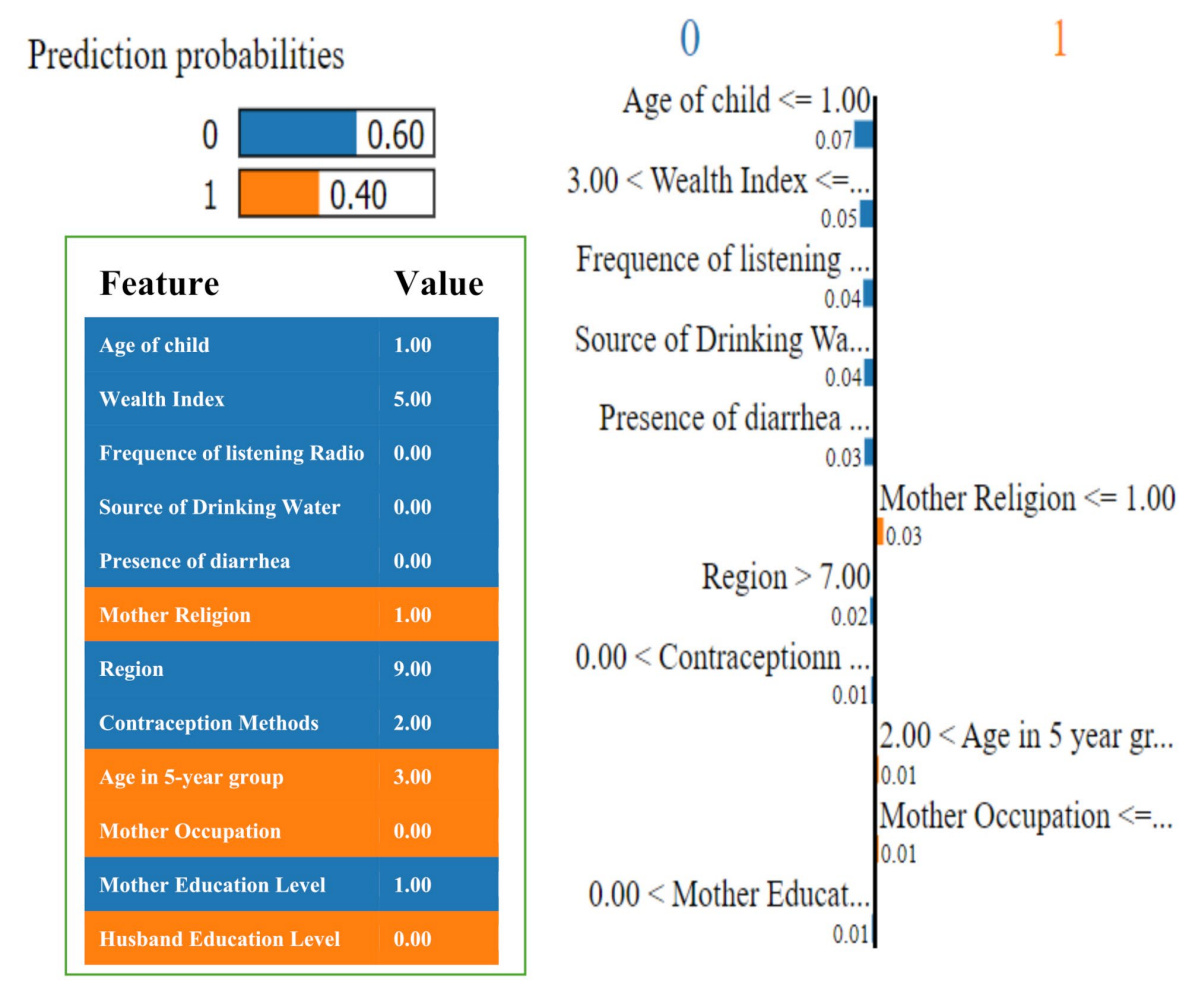
Visualization of the local contribution of sociodemographic features through the LIME model in classifying a single test instance (predicted class = MDD (0)) using the CatBoost model

As illustrated in Figs. 3 and 4, the LIME model is utilized on the data to ascertain how a model predicts and elucidates the contribution of attributes to the prediction of DD using the LightGBM and CatBoost models, respectively.

In Fig. 3, the LIME visualization is presented for the LightGBM model predicting the level of DD among preschool children with MDD, with a predicted probability of 72% for the test MDD instance. The five most significant features of this model are the child’s age, wealth index, region, source of drinking water, and mother’s education level. The feature importance for these five attributes is 13%, 6%, 6%, 5%, and 3%, respectively. Figure 4 illustrates the CatBoost model’s ability to predict the level of DD among preschool children through LIME visualization, with a predicted probability of 60% for the test MDD instance. The five most significant features of this model are the child’s age, wealth index, media coverage (frequency of listening to the radio), source of drinking water, and presence of diarrhea. The feature importance for these five attributes is 7%, 5%, 4%, 4%, and 3%, respectively. The child’s age and wealth index are common attributes that significantly contribute to predicting the level of DD using both the LightGBM and CatBoost models.

## Discussion

In this research paper, the ML approach was used to determine the level of DD in preschool children. A total of 28,047 instances before applying SMOTE techniques were used in the dataset. For feature selection, mutual information, the chi-square test, and step-backward feature selection were used. Two XAI approaches were used to improve our understanding of the results of the ML algorithm. The LightGBM model performed better than the other ML models in terms of accuracy classification metrics. This indicates that the best-suited model for predicting the DD of preschool-aged children with MDD and those with HDDs is the most accurate and reliable. According to our ML findings, the Eli5 interpretable model described region, wealth index, and age in the 5-year group, age of child, and husband education level with greater weight for predicting the DD of preschool children in Ethiopia using the LightGBM model.

Several studies have used different approaches for better predicting DD in preschool children as illustrated in Table 6. Multivariate logistic regression analysis was utilized [1, 6, 24] to identify factors associated with DD among preschool children using a cross-sectional survey design with the help of multivariate logistic regression analysis, and the age of preschool children, household wealth index, and maternal education level were among the most significantly associated features with the dietary diversity of preschool children. Our findings support this finding. By utilizing bivariable analysis for multivariable analysis to identify independent determinants of dietary diversity [2] Mothers’ education, mothers currently working, mothers’ wealth index, and the number of children under five years of age were significantly associated with preschool-aged children with MDD. The results of this study also supported our findings; mothers’ education level, wealth index, and age of a child were among the most significant features associated with the level of preschool DD based on the results of the XAI approach. In Bangladesh, a logistic regression, random forest, decision tree, support vector machine (SVM), K-nearest neighbor, gradient boosted tree and naïve Bayes methods were used to predict the factors influencing MDD outcomes via statistical analysis in combination with ML algorithms. The random forest algorithm achieved the best performance, with an accuracy of 85.4%, compared with the other machine learning models [25]. Table 6 illustrate the comparison of existing and proposed methodology for the prediction model.

**Table 6:** Comparison between existing models and our proposed methodology

No	Paper	Design of Study	Method Used	Explainable AI Approach
1.	[7]	Community-based cross-sectional study	Multivariable logistic regression model No ML Approach	No
2.	[26]	Institutional-based cross-sectional study	Multivariate logistic regression No ML Approach	No
3.	[28]	Community-based cross-sectional study	Multivariable logistic regression model No ML Approach	No
4.	[29]	Experimental study	ML approach	No
5.	[30]	A community-based cross-sectional study	A binary logistic regression was fitted to identify significant factors associated with MDD No ML Approach	No
6.	[31]	Experimental design	Multilevel logistic regression analysis No ML Approach	No
7.	Our proposed model	Experimental design	Ensemble ML approach	LIME, ELI5

## Conclusion

Dietary diversity (DD) is crucial for improving food intake across different food groups; however, it continues to pose a significant challenge for impoverished populations in the developing world. This study investigates the application of explainable machine learning (ML) methods to predict dietary diversity in preschool children. We developed an explainable ensemble ML model that captures the intricate relationships and interactions among various sociodemographic factors influencing DD. Our methodology encompassed data collection, preprocessing, feature selection, and model construction, utilizing a dataset of 63,651 instances with 16 attributes, enhanced through the synthetic minority oversampling technique. In identifying MDD, our interpretable models Eli5 and LIME identified the child’s age and the household wealth index as the most significant predictors. We utilized three feature selection methods: mutual information, the chi-square test, and the step-backward feature selection algorithm, with the latter demonstrating the highest effectiveness.

To evaluate the performance of our ensemble ML model, we utilized various assessment metrics, including accuracy, precision, recall, F1-scores, and ROC analysis. The Light Gradient Boosting model reached a peak accuracy of 95.3%. Key variables identified for predicting MDD in Ethiopia included the child’s age, household wealth index, region of residence, source of drinking water, frequency of media exposure (such as radio), and the mother’s education level. These findings can guide nutritionists and policymakers in developing targeted initiatives to enhance dietary diversity and improve nutritional outcomes for preschool children. Overall, the predictive model developed serves as a valuable tool for decision-making and resource allocation in the field of nutrition, aiding in guiding interventions that can significantly influence preschool children’s dietary diversity.

## Data Availability

Data for this study were sourced from Demographic and Health. surveys (DHS) and are available at https://www.dhsprogram.com.
